# A Malware Detection Scheme Based on Mining Format Information

**DOI:** 10.1155/2014/260905

**Published:** 2014-06-02

**Authors:** Jinrong Bai, Junfeng Wang, Guozhong Zou

**Affiliations:** ^1^College of Computer Science, Sichuan University, Chengdu 610065, China; ^2^School of Information Technology and Engineering, Yuxi Normal University, Yuxi 653100, China

## Abstract

Malware has become one of the most serious threats to computer information system and the current malware detection technology still has very significant limitations. In this paper, we proposed a malware detection approach by mining format information of PE (portable executable) files. Based on in-depth analysis of the static format information of the PE files, we extracted 197 features from format information of PE files and applied feature selection methods to reduce the dimensionality of the features and achieve acceptable high performance. When the selected features were trained using classification algorithms, the results of our experiments indicate that the accuracy of the top classification algorithm is 99.1% and the value of the AUC is 0.998. We designed three experiments to evaluate the performance of our detection scheme and the ability of detecting unknown and new malware. Although the experimental results of identifying new malware are not perfect, our method is still able to identify 97.6% of new malware with 1.3% false positive rates.

## 1. Introduction

While the computer application technology develops at full speed, the security of computer system makes people worried. The security of the computer system becomes more vulnerable than ever and faces more security threats because the technological progress brings the complexity of the application environment. Computers in the network cannot be protected due to the open characteristic of the internet and its lack of central control and global view. Under this network environment, malware has stronger reproduction and destructive ability. Malware is “any code added, changed, or removed from a software system to intentionally cause harm or subvert the system's intended function” [1]. Although researchers have already done lots of work on malware detection, the current malware detection technology still has very significant limitations.

A majority of antivirus vendors deploy signature based malware detection techniques that utilized predefined signatures' set (signature is unique hex code strings in each malware or infected files). Its procedure is as follows: (1) a new malware starts propagating on a large scale, (2) forensic experts of antivirus companies get its samples to study its behavior, (3) they assign it a signature which is effectively a sequence of its instructions, (4) the signature is added to the database, (5) customers are notified to update their signatures' set, and (6) customers update their signatures' set and are finally protected against this malware. It suffers from three main shortcomings: (1) it is unable to detect new malware and unknown malware, (2) the size of the signatures' set and the time of matching signatures will increase exponentially, and (3) during the period between the appearance of a new (unknown) malware and the update of the signatures' set of the anti-virus clients, millions of computers are vulnerable to the new malware. Moreover, malware writers often use sophisticated packing techniques to evade signatures and the advent of more sophisticated malware writing techniques such as polymorphism and metamorphism makes it even harder to detect a malware. Polymorphic and metamorphic malware change their appearance every time when they propagate.

Spinellis [2] has proved that the detection of bounded-length viruses is an NP-complete problem. Therefore, we should use a variety of heuristic knowledge to identify malware and improve the accuracy of judgments. A number of nonsignature based malware detection techniques have been proposed recently. These techniques analyze several features of malware, such as machine-level code, disassembled code, static calls from the disassembled code, and run-time API (application programming interface) calls. The common bottlenecks of such techniques are their high false positive rate and large processing overheads.

The format information of binary executable is affected by its behavior. Binary executable follows basic format, such as PE (portable executables) and ELF (executable and linking format). It consists of a number of headers and sections that tell the OS (operating system) loader how to map the file into memory. Malware and infected executable also follow the format, but they have many differences with the benign executable in format information. In this paper we presented a detection scheme which overcomes the limitations of current malware detection techniques that uses format information of PE files to efficiently detect zero-day (i.e., previously unseen) malicious executables. Based on in-depth analysis of the static format information of PE files, we extracted 197 features from format information of PE files. These features were extracted from headers of all major portions of PE files and many of them do not contribute to the classification accuracy (or even diminish it). We applied feature selection methods to reduce the dimensionality and enhance compactness of the features. When selected features were trained using classification algorithm; the results of our experiments indicate that our scheme achieves about 99% detection rates with less than 1.4% false positive rates for distinguishing between benign software and malware. The most important is that our method can identify the unknown and new malware.

The rest of the paper is organized as follows. In Section 2 we discuss related work and motivation for our approach. In Section 3 we briefly describe PE format and discuss format differences between benign software and malware.  Section 4 presents the architecture of our approach.  Section 5 discusses our experiments. In Section 6 we analyze the experimental results that are performed to determine which scheme performs better. Finally we present our conclusions in Section 7.

## 2. Related Works

Although the problem of determining whether unknown software is malicious or not has been proven to be generally undecidable [3], detecting malware with an acceptable detecting rate is still possible. A number of approaches have been proposed to detect unknown malware. Traditionally, there are two main approaches to detect malware: static approach and dynamic approach.

### 2.1. Static Approach

Static approach checks executable binaries or assembly code without actually executing any of the codes in question. A recent survey [4] on applying machine learning classifiers on static features to detect unknown malware suggests that static approach achieves a very high accuracy while maintaining low false positives. The major advantage of static approach over its dynamic counterpart is that it is free from the overhead of execution time.

Schultz et al. [5] presented a data-mining framework to detect new, previously unseen malware and proposed three different methods to detect malicious executables on the Windows platform. The first technique is based on the list of DLLs (dynamic link library), the list of DLL function calls, and the number of different function calls within each DLL used by the binary. In the second technique, they used the string as a binary feature. In the third technique, they used two bytes* n*-gram (instead of a string) as binary feature.

Later, Kolter, and Maloof [6] improved Schulz's third technique, by applying byte* n*-grams instead of nonoverlapping sequences. 4 grams are used as features and top 500* n*-grams are selected through information gain measure. They used naive bayes, instance based learners, decision trees, TFIDF, and support vector machines and also boosted last three classification algorithms. The best result is achieved by boosted J48 at AUC, 0.996.

OpCode (operational code) sequence* n*-grams which were extracted from the files after disassembly are used as representation of the executables in [7–9]. TF and TFIDF of each OpCode* n*-gram were calculated as the weight of features. The evaluation of many factors is conducted based on eight types of classifiers. The experimental results indicate that the use of OpCode* n*-grams achieves better results than the byte* n*-grams. Furthermore, the imbalance problem of datasets was investigated to determine the optimal settings of the training set for each classifier.

In order to label suspicious files to malware or benign software, information security experts need to analyze manually suspicious files and it is a time-consuming task. Nissim et al. [10] presented an AL (active learning) framework and introduced two new AL methods to acquire new malwares. The results indicate that their method outperforms random selection for all performance measures and can assist antivirus vendors to improve efficiency.

### 2.2. Dynamic Approach

Dynamic approach aims to test and evaluate a program by actual execution of code in real time and it is significantly less vulnerable to code obfuscating transformations.

Stopel et al. [11] employed ANN (artificial neural networks) to detect the presence of computer worms based on the computer's behavioral measures, and sixty different parameters of the infected computers were measured. The results indicate that this method has computational advantages and can detect previously unknown worms. Stopel et al. [12] improved worm detection with ANN through three temporal analysis preprocessing techniques and reduced the number of features significantly by using various feature selection techniques. The results show that preliminary temporal processing does not increase the detection accuracy significantly, and the accuracy of worm detection may increase when only the most important features are used.

Moskovitch et al. [13] applied data mining to detect unknown computer worm's activity based on behavior features. Several computer configurations and background applications activity are used to test this new method. The results indicate that an above 90% average accuracy is achieved using just 20 features, and the influence of the environment is not obvious for worm detection. Furthermore, behavior features with machine learning techniques are also used in host based intrusion detection [14, 15].

Shabtai et al. [16] presented eDare framework to address the risks stemming from malicious software propagating via networks. Network-based traffic was extracted and various types of algorithms were employed to detect unknown malware in eDare framework. Static and behavioral analyses of malware were incorporated in this system which also presented novel automatic signature generation algorithm.

Ahmed et al. [17] introduced a malware detection technique combining two different dynamic features (from spatial and temporal information) available in run-time API. Park et al. [18] used run-time execution of malware files to generate maximal common subgraph to detect malware. Tian et al. [19] proposed an automated tool running in a virtual environment to extract API call features from executables and apply statistical methods and pattern recognition algorithms to differentiate between benign software and malware.

Dynamic approach is time-consuming as each malware sample must be executed for a certain time period and analysis environment is quite different from a real run-time environment. Furthermore, the main limitation of dynamic analysis is that typically only a single execution path is examined.

### 2.3. Motivation for Our Approach

Although the above approaches show good results, these detection techniques are limited in that these are blind to the format of the program which carries important information to understand its behavior. The idea of using format features in malware detection has also been explored by previous works. Szor [20] summarized format abnormalities of the malware and infected file and discussed some heuristic detection method based on format abnormalities. Weber et al. [21] used format features of executables that are likely to indicate the presence of inserted malicious code. Dai [22] reproduced Kolter and Maloof's [6] experiment, analyzed filtered features, and found that most of the features are the format features of PE file and very few are OpCode sequences. Those works used the static format abnormalities as heuristic malware detection rules, but none of these works employed data mining to address the problems. Data mining is used to detect unknown malware in recent years [5–17] and these studies achieve high accuracy and low false positive rate. Shafiq et al. [23] presented PE-miner framework which mined PE file header to identify unknown malware. Later, Shafiq et al. [24] leveraged packer detection to improve this method. Compared with previous studies [23, 24], our approach extracted different features from PE file header, employed different feature selection methods, and designed three experiments to evaluate the ability of detecting unknown and new malware.

## 3. Overview of PE Format

PE stands for portable executable. It is the native executable format of Win32. Its specification is derived somewhat from the Unix COFF (common object file format).

**Figure 1:**
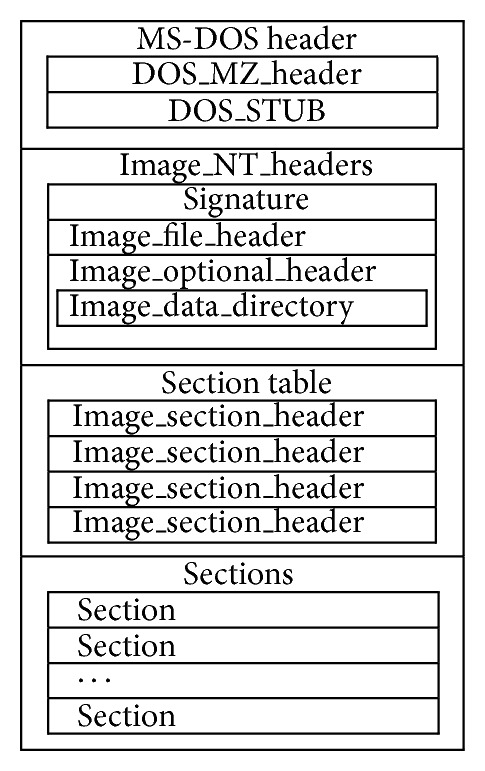
The PE format.

The format information of PE file is illustrated in Figure 1. It is basically a data structure that encapsulates the information necessary for the Windows OS loader to manage the wrapped executable code. A PE file consists of a PE file header and a section table (section headers) followed by the sections' data. The PE file header consists of a MS DOS header, the PE signature, the image file header, and an optional header. The file headers are followed immediately by section headers. Section header provides information about its associated section, including location, length, and characteristics. Section is the basic unit of code or data within a PE or COFF file. Different functional areas, such as code and data areas, are separated logically into sections. In addition, an image file can contain a number of sections, such as.tls and.reloc, which have special purposes.

Many fields of PE file have no mandatory constraint. There are a number of redundant fields and spaces in PE file, so that it has created opportunities for malware's propagation and hide. It also makes the format information of malware and benign software show many differences. What is below describes particular format problems unlikely to happen in PE programs compiled with a 32-bit compiler (such as Microsoft and Borland compiler) [20]: (1) code execution starting in the last section, (2) suspicious section characteristics, (3) suspicious code redirection, (4) suspicious code section name, (5) the entry point not pointing into any of the sections, (6) import address table not patched, (7) multiple PE header, (8) incorrect size of code in header, and so forth. Although format information is not very advanced, it is an effective way to detect even polymorphic malware.

In summary, there are many differences in format information between malware and benign software. The application of data mining methods to study these differences of format information is a feasible way to detection of known and unknown malware.

## 4. System Architecture

**Figure 2:**
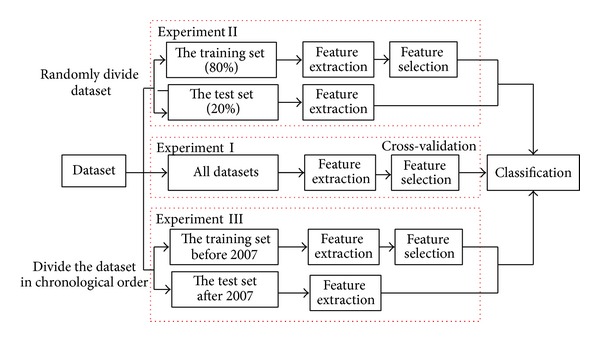
The architecture of our method.

As shown in Figure 2, we designed three experiments to verify the performance of our detection scheme. In Experiment I, we followed the experiment process used universally in previous literature and tenfold cross-validation was used to evaluate experimental result. Because feature selection was performed in the whole dataset, it may lead to overfit experimental data. Dataset was randomly divided into the training set and the test set into Experiment II, and feature selection and training classifier were conducted in the training set. Experiment II ensured that our approach can detect unknown malware and not overfit experiential data. When old and new malware are mixed in the training set, the ability of detecting new malware cannot be evaluated objectively. In order to evaluate the performance of identifying new malware, we partitioned dataset to the training set and the test set by chronological order in Experiment III. The process of Experiment III is similar to Experiment II.

Three experiments consist of three main common modules: (1) feature extraction, (2) feature selection, and (3) classification. We discussed each module separately. Feature extraction module extracts format information of dataset. A training set of benign software and malware is provided to the system. Each file is then parsed, and features representing each file are extracted based on format information. Afterwards, the feature selection module ranks these features to filter redundant features or features with less classification potential. The features are extracted from the test set based on filtered features of the training set. As a result, the training set and the test set serve as input for a classification algorithm (such as a decision tree or random forest). Classification module eventually classifies the executable as malicious or benign.

## 5. Experiments

### 5.1. Dataset

Dataset is divided into malware and benign software. We should obtain enough representative training dataset in actual antivirus software. Because we just verify the feasibility of our method, we collected 8592 types of benign software and 10521 types of malware. All are in the Windows PE format. We obtained benign software from Windows folder and Program Files folder and used commercial software to verify that each executable was indeed benign. We obtained malware from the website VXHeavens [25]. The distribution of malware is in Table 1.

### 5.2. Feature Extraction

There are many format features in PE files, but most of those features are not helpful in distinguishing malware and benign software. Based on our empirical studies and in-depth analysis of the format features of the PE files, we extracted 197 features that have the potential to distinguish between benign software and malware, from given PE files. These features are summarized in Table 2. In the below discussion, we gave a brief description of the extracted features in our study.

#### 5.2.1. DLLs Referred and APIs Referred

We predicted the behavior and functionality of an executable program by analyzing its usage of the DLLs and Win32 APIs. We dumped each executable file's import table of the PE format and counted all the DLLs and APIs that are used. Removing those DLLs and APIs appeared less than 100 times; there are 46 different DLLs and 597 different APIs left. We computed information gain for each DLL and API and selected the best 30 different DLLs and 30 different APIs according to the information gain.

#### 5.2.2. The Number of DLLs Referred, APIs Referred, and Exported Symbols

The imports table lists all of the symbols that need to be resolved and imported at load time from other DLLs. Moreover, Windows also allows programs to load and unload DLLs explicitly using LoadLibrary and FreeLibrary and to find addresses of symbols using GetProcAddress. Most types of malware use the latter approach, so that the number of symbols in their imports table is relatively fewer than that of benign software. The exports table contains information about symbols that other PE files can access through dynamic linking. Exported symbols are generally found in DLL file and most types of malware do not have exported symbols.

#### 5.2.3. PE File Header

The PE file header contains general information that is useful for loading and running an executable file. Experimental result is possibly misled by some features of PE file header such as type of the machine, linker version, and the operating system version which is the same in benign software and has the obvious difference with malware. Including these features, we would get a higher accuracy, so that we excluded those useless features and extracted all the rest.

#### 5.2.4. Section Headers

Section header provides information about its associated section. In this study, we only considered text, data, rsrc, rdata, and reloc sections because they are commonly present in the executables.

#### 5.2.5. Resource Directory Table

Most Windows executables contain resources, a general term that refers to objects such as cursors, icons, bitmaps, menus, and fonts. A PE file can contain a resource directory for all of the resources the program code in that file uses. We extracted the number of 23 kinds of resources type. Malware types rarely uses graphical resources, so the total number of their resources is relatively fewer than that of benign software.

### 5.3. Feature Selection

The objective of feature selection is threefold: improving the prediction performance of the classifier, providing faster and more cost-effective classifier, and gaining a deeper insight into the underlying processes that generated the data. Feature selection methods can be divided into two main categories according to the dependence on the classifiers: filter approach and wrapper approach.

In the wrapper approach, the feature subset selection is done by target learning algorithm as a black box. The feature subset selection algorithm conducts a controlled enumeration search for a good subset using the target learning algorithm itself as part of the evaluation function. The filter approach selects features independent of the target learning algorithm. The filter approach tries to select a feature set with a predefined evaluation criterion such as *t*-test, *χ* ^2^-test, and information gain.

We applied the CfsSubsetEval (filter approach) and the WrapperSubsetEval (wrapper approach), which are implemented in WEKA [26] to select the most effective feature subset from 197 features.

### 5.4. Classification

Data mining has been the focus of many malware researchers in the recent years to detect unknown malwares. The particular problem of labeling a program as malware or benign software is an example of the general classification problem. A number of classifiers have been built and shown to have a very high accuracy rate.

We applied several classification learning methods, which are implemented in WEKA [26]: J48 (decision tree) [26] and random forest [27]. We also employed ensemble methods (boosting [28] and bagging [29]) to improve performance of J48.

## 6. Experimental Result and Analysis

### 6.1. Evaluation Description

For evaluation purposes, the following classical measures are usually employed. The TPR (true positive rate) measure is the rate of positive instances (i.e. malware files) classified correctly. FPR (false positive rate) is the rate of negative instances (i.e., benign files) misclassified. The total accuracy measures the number of absolutely correctly classified instances, either positive or negative, divided by the entire number of instances.

The ROC (receiver operating characteristic) curve is a graph produced by plotting the fraction of TPR versus the fraction of FPR for a binary classifier as its discrimination threshold varies. We also used the AUC (area under the ROC curve) measure in the evaluation process.

### 6.2. Experimental Results I

In Experiment I, tenfold cross-validation was used to divide the whole dataset to the training set and the test set. We used the subset selected by filter approach as features and employed four classification algorithms (J48, random forest, bagging (J48), and AdaboostM1 (J48)) to train classifier. Detection accuracy rate of trained classifiers is about 99%. We used the subset selected by wrapper approach as features and employed random forest classification algorithm to train classifier. Detection accuracy of trained classifiers is 99.1%. The results of all experiments are presented in Table 3.

### 6.3. Results Analysis

As can be seen from Table 3, all classification algorithms detected about 99% of unknown malware and the relative performance of those classification algorithms is roughly the same. We can see that two ensembles (AdboostM1 and bagging) performed slightly better than J48. More specifically random forest performed slightly better than other classification algorithms. Because wrapper methods applied the classification algorithm that will be used to generate the final classifier to test the performance of each feature subset, the performance of the wrapper approach is better than that of the filter approach.

Table 3 shows the AUC results of all classification algorithms. We can see that the AUC results of all classification algorithms are above 0.994, all methods performed comparably and the AUC value of two classification algorithms (AdboostM1 (J48) and random forest) is 0.998 which is very closer to the AUC value of the best possible classifier.

### 6.4. Selected Feature Analysis

Table 4 presents the mean values of the selected feature used in our study and these features are ordered according to the contribution to the classification task. As we can see, most types of benign software have a large number of entries in the import address table because they have complex functions and import different Windows API functions from the import address table. Most types of malware load and unload DLL explicitly using LoadLibrary and FreeLibrary in order to hide their malicious purpose. The mean values of API functions such as lstrlenW, __adjust_fdiv, GetModuleHandle, CreateFileW, _initterm, and RegDeleteKey in benign software are far greater than that in malware. But there is one notable exception. 25.9% of malware imports wsock32.dll, while only 1.6% of benign software imports this DLL. It explains that most types of malware carry out propagation and damage through network. Mscoree.dll file is a part of Microsoft.Net and contains coded programs that are used by the framework for running programs. 18.9% of benign software imports mscoree.dll, but only 0.2% of malware imports this DLL. The reason is that malware which is developed using Microsoft.Net framework will greatly increase its size and depend heavily on this framework. So few types of malware are developed using Microsoft.Net framework. The imm32.dll, also known as the input method manager, helps minimize the effort needed by users to enter text. Few types of malware import this DLL, because malware rarely presents a list of candidate characters which the user may want.

The mean values of several features in the PE file header have obvious differences between malware and benign software. The.debug section is used to contain compiler-generated debug information, and the SizeOfDebugData is the size of debug information. The debug data of malware is significantly less than that of benign software because malware reserves less debug data in order to prevent debugging. The NumberOfSymbols indicates the number of entries in the symbol table. The benign software preserves the symbol information in the.debug section, while malware rarely has the.debug section and reserves the symbol information in the symbol table. So the number of symbols in malware is far greater than that in benign software. The characteristics field indicates that the file is an executable image or a dynamic-link library. The ImageBase field specifies the preferred address of the first byte of image when loaded into memory. The default for dynamic-link library is 0x10000000, and the default for executable image is 0x00010000. The Dll characteristics indicate whether a DLL image includes entry points for process and thread initialization and termination. Most types of malware are executable image, while parts of benign software are dynamic-link library. So the mean values of characteristics, ImageBase, and Dll characteristics have obvious differences between malware and benign software.

In the directory table, the mean size of certificate table (certificate table is used to associate verifiable statements with a file by a software manufacturer) in benign software is far greater than that of malware. The reason is that most types of benign software have certificate table while most types of malware do not have. The load configuration table includes reserved SEH technology which provides a list of safe structured exception handlers that the operating system uses during exception dispatching. The mean value of size of load configuration table in malware is far greater than that of benign software.

The characteristics of four sections (.text,   .rsrc, .reloc, and  .rdata) in malware are distinguished from that in benign software. The characteristics of section specify whether the section is readable, writeable, and executable and so on. The characteristics of code section in malware have writeable exception and the characteristics of data section and resource section in malware have writable and executable exception.

Moreover, the malicious executables have fewer entries in resource section—such as message table, group icon, and version—than those in benign software. 95% of benign software has version information, but only 40.8% of malware has version information.

### 6.5. Experimental Results II

Most of malware detection approaches which apply data mining techniques have a common problem: there is no intersection between the training set and the test set during classification experiment, but those approaches select feature from the whole dataset. It leads to overfit experiment data and achieves better experiment result. We conducted another experiment. The entire sample set is randomly divided into two parts: the training set (80%) and the test set (20%). We used only the training set to select feature and train the classifier and then used the test set to test the trained classifier. All experimental results are shown in Table 5.

Most of malware detection methods which select feature only from the training set could significantly reduce the detection accuracy rate. It can be seen from Table 5 that only J48 classification algorithm fell slightly; on the contrary, other classification algorithms increase slightly. There is no significant effect on the experimental results when our approach selects feature only from training set. Compared with other detection methods using data mining techniques, our detection method is more robust.

### 6.6. Experimental Results III

Most of malware detection approaches claim that their methods can detect new malware. But these approaches are evaluated by cross-validation and old malware and new malware are mixed in the training set. In this way, it cannot validate the ability of identifying new malware for these methods. Chronological evaluation is introduced firstly in [6, 30]. In order to evaluate whether our scheme can detect new malware with high accuracy, we divided the dataset into the training set and the test set in chronological order. Malware which appeared before 2007 is added to training set, and the test set contains malware which was found after 2007. The benign software is also partitioned by chronological order. The training set contains 4361 types of malware and 3886 types of benign software, and the test set consists of 6060 types of malware and 4706 types of benign software. The results of all experiments are presented in Table 6.

As can be seen from Table 6, the performance of all classification algorithms fell obviously. J48 classification algorithm has serious performance degradation, and its AUC value is only 0.928. We can conclude that the performance of J48 algorithm is not stable and it may overfit the training set. The good news is that the random forest and the Adaboost (J48) achieve acceptable high accuracy rate and their AUC value is above 0.993. Although the experimental results are not perfect, our method is still able to identify 97.6% of new malware with 1.3% false positive rates. To sum up, identifying new malware is still a challenging problem and we plan to improve our approach in our future work.

### 6.7. Comparisons among Three Experiments

**Figure 3:**
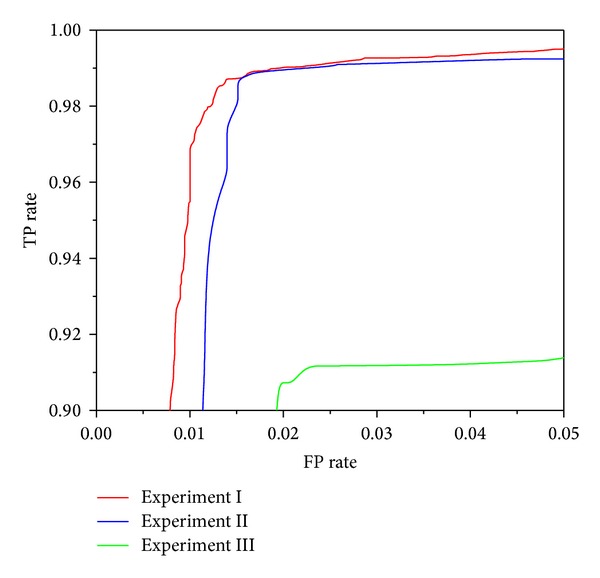
ROC curves for J48 classification algorithm.

**Figure 4:**
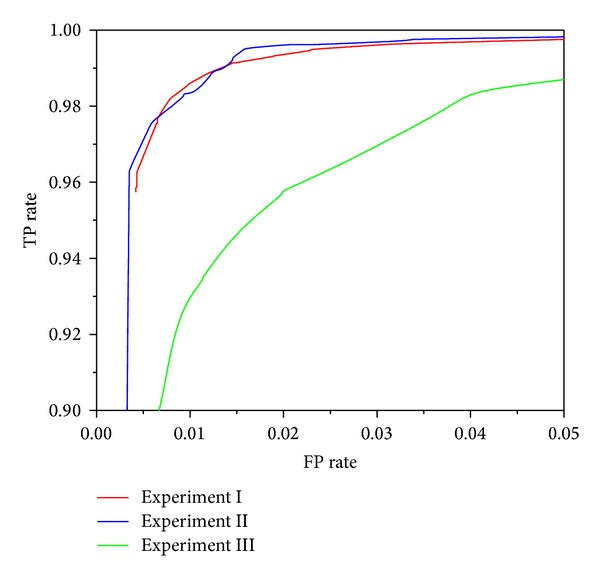
ROC curves for random forest (filter) classification algorithm.

**Figure 5:**
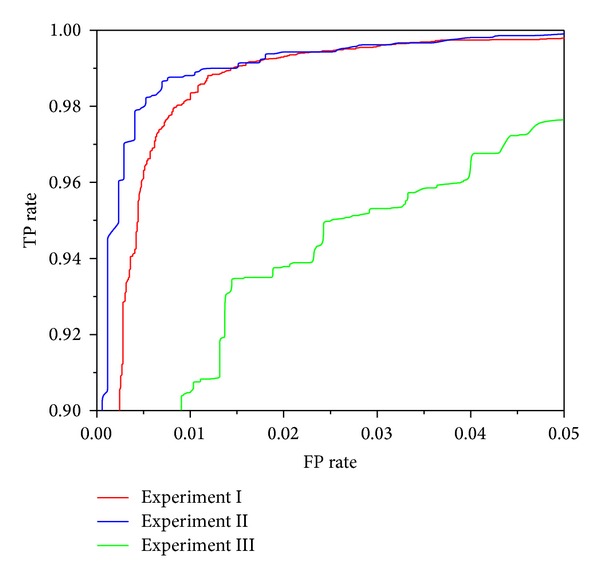
ROC curves for Adaboost (J48) classification algorithm.

**Figure 6:**
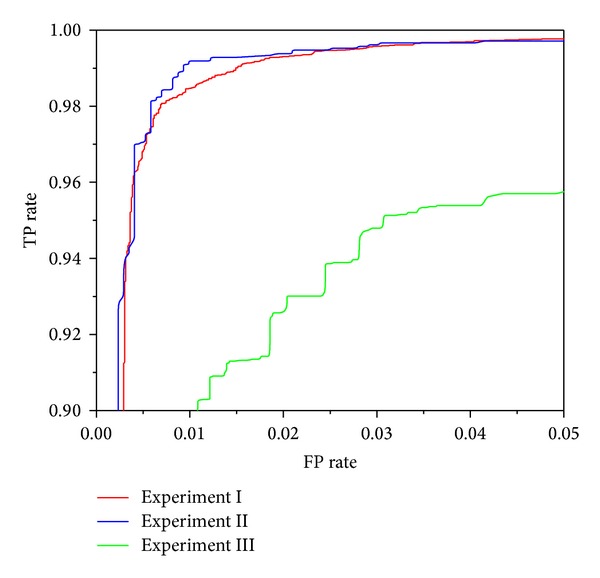
ROC curves for bagging (J48) classification algorithm.

**Figure 7:**
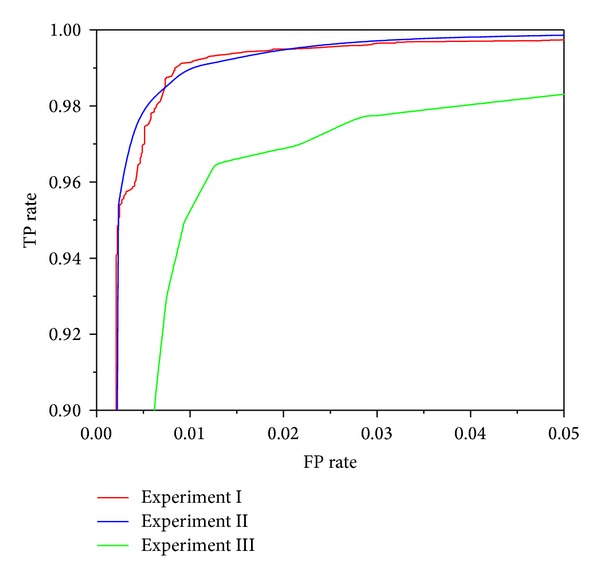
ROC curves for random forest (wrapper) classification algorithm.

Figures 3, 4, 5, 6, and 7 show the ROC curves of all classification algorithms in three experiments. We can see that the ROC curve for random forest algorithm which used the result of wrapper approach dominated all others. Two ensemble algorithms (AdboostM1 (J48) and bagging (J48)) which used the result of filter approach performed comparably and J48 algorithm did not perform as well as all others. To sum up, wrapper approach achieved better results than filter approach.

In Experiment II, it can be seen from Figures 3–7 that ROC curve for J48 classification algorithm fell slightly and the ROC curve for other classification algorithms does not change obviously. In Experiment III, We can see from Figures 3–7 that the performance of all classification algorithms drops significantly and the performance of the random forest algorithm is relatively stable.

Through comparing three experiments, a conclusion is drawn that our approach can detect unknown malware with high accuracy and can identify new malware with acceptable accuracy while maintaining low levels of false positive rate. J48 classification algorithm is not stable and ensemble methods (boosting and bagging) improve obviously the performance of J48 algorithm. The random forest algorithm dominates other classification algorithms and is stable and reliable.

### 6.8. Comparisons with the Related Static Methods

Our method is compared with related static methods mainly from the accuracy, detection time, and validity.  Table 7 tabulates the number of features, the accuracy, and AUC for our method, KM [6], OpCode [9], and PE-Miner [23]. A macrolevel scan through the table clearly shows that our method is slightly better than other methods. Our method selected less and effective features and achieved similar accuracy and AUC. Because PE-Miner did not make mixed types of malware experiments; their results are better than the actual results.

In terms of detection time, signature-based method needs to traverse the suspicious executable file and match content with huge signature database. The KM method needs to convert the suspicious executable file to hex file and traverse hex file to generate features. Furthermore, 500 features are used to train classifier in the KM method and the trained classifier is more complex. The OpCode method first disassembles the suspicious executable file and the disassembled file is traversed to generate features. It takes more time to disassemble the executable file, and the number of features in this method is more than that of our method. Our method does not need to traverse the suspicious executable file and only needs to parse the PE header. The number of features in our method is less than that of KM, OpCode, and PE-Miner methods. In summary, it takes less time to detect malware in our method than the above methods.

Compared with our method, signature-based antivirus software cannot detect unknown malware. The OpCode approach is not always feasible because some executable files cannot be disassembled properly. Because most types of malware use executable packing and obfuscation techniques in order to hide their malicious code and evade detection, KM and OpCode approaches can only identify a portion of malware variants. But malware which employs packing and obfuscation techniques still complies with the constraints of the PE format and our detection method is still valid. Because the code section of PE file is not inspected in our method, we will make more experiments to confirm the validity of our method in our future work.

## 7. Conclusion

In this paper, we have proposed a malware detection approach by mining format information of PE files and described experiments conducted against recent Win32 malware. The new detection approach makes possible the highly accurate detection of unknown and new malware, based on previously seen samples, while maintaining low levels of false positive rate. Experimental results indicate that the accuracy of the top classification algorithms is 99.1% and applying ensemble algorithms improves classification accuracy substantially. We compared the different feature selection methods and concluded that wrapper approach achieves better results than filter approach. Compared with other static approaches, our method has the slightly better performance in the accuracy, detection time, and validity. The main shortcoming of our method is that crafty malware writers could forge the PE header of malware which is similar to a benign one to evade detection.

## Figures and Tables

**Table 1:** The distribution of PE format malware.

Malware categories	Backdoor	Constructor + Virtool	DoS + Nuker	Flooder	Exploit + Hacktool	Worm	Trojan	Virus	Total
Quantity	3184	418	254	339	395	1422	3012	1497	10521

**Table 2:** List of the features extracted from PE files.

Feature description	Type	Quantity

DLLs referred	Integer	30
APIs referred	Integer	30
The number of DLLs referred	Integer	1
The number of APIs referred	Integer	1
The number of sections	Integer	1
The number of symbols in export table	Integer	1
The number of items in reloc section	Integer	1
Dos header—e_lfanew	Integer	1
IMAGE_FILE_HEADER	Integer	5
IMAGE_OPTIONAL_HEADER	Integer	16
IMAGE_DATA_DIRECTORY	Integer	32
.text section—header field	Integer	11
.data section—header field	Integer	11
.rsrc section—header field	Integer	11
.rdata section—header field	Integer	11
.reloc section—header field	Integer	11
Resource directory table and resources	Integer	23

Total		197

**Table 3:** Experimental results for all classification algorithms (feature selection from the whole dataset).

Feature selection method	The number of features	Algorithm	TPR (%)	FPR (%)	Accuracy (%)	AUC

Filter	19	J48	98.9	1.4	98.7	0.994
		Random forest	99.1	1.4	98.9	0.996
		Adaboost (J48)	99.0	1.0	99.0	0.998
		Bagging (J48)	98.9	1.3	98.8	0.997

Wrapper	20	Random forest	99.1	1.0	99.1	0.998

**Table 4:** The mean values of selected features.

Name of feature		Mean values	
Features selected by CfsSubsetEval	Features selected by WrapperSubsetEval	Malware	Benign software
SizeOfDebugData	SizeOfDebugData	0.982	22.6
ImageBase	ImageBase	1.7 × 10^7^	7.6 × 10^8^
SizeOfCertificateTable	SizeOfCertificateTable	8.87	2.8 × 10^3^
DllCharacteristics	DllCharacteristics	29.8	3.6 × 10^3^
.reloc.characteristics	.reloc.characteristics	7.4 × 10^8^	9.3 × 10^8^
SizeOfLoadConfigurationTable	—	1.0 × 10^5^	22.7
NumberOfVERSION	NumberOfVERSION	0.408	0.95
Characteristics	Characteristics	1.5 × 10^4^	7.1 × 10^3^
—	GetModuleHandle	0.001	0.23
AddressOfDebugData	—	3 × 10^3^	1.4 × 10^5^
lstrlenW	—	0.005	0.296
DisableThreadLibraryCall	—	0.003	0.253
.rsrc.characteristics	—	1.4 × 10^9^	1.0 × 10^9^
—	CreateFileW	0.002	0.204
—	_initterm	0.036	0.416
—	RegDeleteKey	0	0.1
__adjust_fdiv	—	0.035	0.415
mscoree.dll	mscoree.dll	0.002	0.189
NumberOfGROUP ICON	—	0.879	1.204
—	BaseOfCode	7.3 × 10^4^	7.8 × 10^3^
wsock32.dll	wsock32.dll	0.259	0.016
—	.text.Characteristics	1.4 × 10^9^	1.5 × 10^9^
—	.rdata.Characteristics	6.2 × 10^8^	3.5 × 10^8^
NumberOfMESSAGE TABLE	—	0.002	0.057
NumberofAPIs	NumberofAPIs	65.1	97.1
SizeOfHeapReserve	—	1.5 × 10^6^	1.0 × 10^6^
—	imm32.dll	0.001	0.013
—	NumberOfSymbols	3.1 × 10^6^	15.9
—	Numberofsections	4.8	4.1

**Table 5:** Experimental results for all classification algorithms (feature selection only from the training set).

Feature selection method	The number of features	Algorithm	TPR (%)	FPR (%)	Accuracy (%)	AUC
Filter	18	J48	98.7	1.5	98.6	0.988
		Random Forest	99.2	1.5	98.9	0.997
		Adaboost (J48)	99.1	1.5	98.8	0.999
		Bagging (J48)	99.1	1.0	99.1	0.998

Wrapper	19	Random Forest	99.1	1.3	99	0.998

**Table 6:** Experimental results for all classification algorithms (the datasets are divided into the training set and the test set in chronological order).

Feature selection method	The number of features	Algorithm	TPR (%)	FPR (%)	Accuracy (%)	AUC
Filter	20	J48	90.7	2.1	94.6	0.928
		Random forest	94.7	1.5	96.7	0.993
		Adaboost (J48)	93.1	1.4	96.0	0.993
		Bagging (J48)	91.3	1.5	95.1	0.969

Wrapper	20	Random forest	96.5	1.3	97.6	0.994

**Table 7:** Comparisons with the related static methods.

Methods	Number of features	Accuracy (%)	AUC
Our method	20	99.1	0.998
PE-Miner	60	—	0.986
KM	500	—	0.996
OpCode	100	96	—
